# The model crisis, or how to have critical promiscuity in the time of Covid-19

**DOI:** 10.1177/0306312721996053

**Published:** 2021-02-16

**Authors:** Warwick Anderson

**Affiliations:** 1 University of Sydney, Sydney, NSW, Australia

**Keywords:** Covid-19, models, crisis, pandemic, AIDS, epidemiology, ecology

## Abstract

During the past forty years, statistical modelling and simulation have come to frame perceptions of epidemic disease and to determine public health interventions that might limit or suppress the transmission of the causative agent. The influence of such formulaic disease modelling has pervaded public health policy and practice during the Covid-19 pandemic. The critical vocabulary of epidemiology, and now popular debate, thus includes R_0_, the basic reproduction number of the virus, ‘flattening the curve’, and epidemic ‘waves’. How did this happen? What are the consequences of framing and foreseeing the pandemic in these modes? Focusing on historical and contemporary disease responses, primarily in Britain, I explore the emergence of statistical modelling as a ‘crisis technology’, a reductive mechanism for making rapid decisions or judgments under uncertain biological constraint. I consider how Covid-19 might be configured or assembled otherwise, constituted as a more heterogeneous object of knowledge, a different and more encompassing moment of truth – not simply as a measured telos directing us to a new normal. Drawing on earlier critical engagements with the AIDS pandemic, inquiries into how to have ‘theory’ and ‘promiscuity’ in a crisis, I seek to open up a space for greater ecological, sociological, and cultural complexity in the biopolitics of modelling, thereby attempting to validate a role for critique in the Covid-19 crisis.

In these trying days, to open with a quotation from Albert Camus may look like a pandemic banality, but when everything else appears unprecedented, the banal surely has merit.^1^ In *La Peste*, Camus (1991) observed that the plague afflicting Oran was a kind of abstraction. ‘Still,’ he wrote, ‘when abstraction sets to killing you, you’ve got to get busy with it’ (pp. 88–89).^2^ Though writing on plague, Camus had been thinking about another crisis, an existential crisis precipitated by war and Holocaust. He was concerned that many humans could summon up nothing but fatigue and inertia in response to such horrors. ‘I know that for a long time already,’ he told readers of American *Vogue*, ‘we humans have been ill at ease in our skins, have been unsure of the future, which is not a normal state for supposedly civilized men’ (Camus, 1946: 86). The crisis pressed upon the world, but most people found themselves overcome with worry, incapable of making decisions or judging the way forward. They were suffering with ‘abstraction and fear’ (p. 86), deluded, unable to work out what to do. And yet, Camus noted, ‘there are among us enough men of decision pledged to do all that is within their power to cure themselves and the world of its present sickness’ (p. 87). Some seventy-five years later, we face another kind of crisis, the Covid-19 pandemic, and still, we grapple with abstraction and fear, and still, we balk at making decisions. How, then, should we come to judgement in the current crisis? How do we get busy with our present sickness?

To the ancient Greeks, the word ‘crisis’ meant discrimination or decision. In the Hippocratic corpus, a crisis was the moment in a disease or fever when its course is determined, a turning point tending either to recovery or death. It implied that a verdict or judgment had been reached. Accordingly, Thucydides (1991; see Cochrane, 1929) deployed the concept of crisis in his description of the plague of Athens to signify the moment of truth in the body politic, a tipping point between success and devastation. Koselleck (2006) suggests that the metaphoric flexibility of crisis – ‘this concept with its inherent demand for decisions and choices’ (p. 358) – allowed it to become a central catchword (*Schlagwort*) in Europe from the seventeenth century onwards. Its everyday clinical significance gradually declined, though lingering in the vague parlance of ‘critical’ condition and ‘critical’ care. Meanwhile, the concept of crisis has come increasingly to frame the body politic, denoting a ‘moment of judgment and diagnosis, as well as the prescription for a therapy’ (Koselleck 2006: 370). Both diagnostic and prognostic, the term conveys the sense of an illness, but also portends an impending transition or else the blocking of radical change. This is what Antonio Gramsci (1971: 275–276) meant when he lamented, ‘The crisis (*la crisi*) consists precisely in the fact that the old is dying and the new cannot be born; in the interregnum a great variety of morbid symptoms (*fenomeni morbosi*) appear.’^3^ Unlike other organic analogies, such as reproductive and life cycles, a crisis is anticipatory, progressive and dynamic. Thus, the concept of crisis, Koselleck (2006: 371) writes, ‘has become the fundamental mode of interpreting historical time’. It is the ‘structural signature of modernity’ (p. 372).

‘In this moment of crisis’, conservative historian François-René De Chateaubriand (1834: 238) realized in the late-eighteenth century, ‘no one can say: “I will do this thing tomorrow”, without foreseeing what tomorrow might bring.’ It is necessary to understand the situation and how it might be evolving: A crisis demands rapid reckoning and then action. Personal or embodied crises thus may call for prognostication and augury, or for psychological inquiry, or for the application of techniques of physical examination and technologies like the thermometer and fever chart. As metaphor and model in the body politic – itself a scaled-up simile, of course – crisis gave rise to historical, sociological and economic explanations, all emerging in the nineteenth century in order to configure one challenge or another.^4^ When epidemic diseases such as cholera swept across Europe and North America in this period, such health crises, magnified across a population, prompted the development of statistical maps and charts in efforts to render them coherent and actionable (Anderson, 2018). Outbreak modelling and epidemic intelligence have become more elaborate and compelling during the past forty years, reaching their apogee in explaining and attempting to manage the Covid-19 crisis. The critical vocabulary of epidemiology, the study of disease patterns, now includes R_0_, the basic reproduction number of the virus (which indicates its transmissibility), and ‘flattening the curve’ and epidemic ‘waves’ – how did this come to be, and what is the function of framing and foreseeing the crisis in this mode? These are issues that concern me here.

Before addressing formulaic modelling mobilized in the Covid-19 pandemic, I want to consider more broadly the functions of the conceptual tools framing a crisis. Starn (1971: 16–17) wrote that ‘what the historian defines as a crisis situation does not necessarily change anything at all so much as reveal the fibre of its subject; it may be something like his [*sic*] best equivalent of the instruments with which the physicist speeds up the processes of matter in order to make them more “visible”.’ The crisis becomes a sort of functional focal point in a structural and organic skeleton. The Berkeley historian was reflecting on the proliferation of crisis talk during the Cold War, much of it involving nuclear flashpoints or youth rebellion or Western civilization or population growth, not emerging infectious diseases. Few in the Global North perceived any crises in the economy or public health in that turbulent era. Even so, ‘notions of crisis cropped up everywhere … in the past two decades’, Starn observed (p. 14). ‘Crisis interpretations have displaced older interpretative scaffoldings’ (Starn, 1971: 19; see also Kramer, 2017; Shank, 2008).^5^ Such sharp framings, so distinct from the *longue durée*, became a means of capturing the deeply felt drama and dynamism of modern historical processes, identifying what seemed to be decisive turning points in the second half of the twentieth century.

‘The use of the concept of crisis’, Koselleck (2006: 37) surmises, ‘is meant to reduce the room for maneuver, forcing the actors to choose between diametrically opposed alternatives.’ Increasingly, this has expedited a reductionist analysis of the situation, along with quantification, standardization of data and statistical modelling of the emerging problem. Defining an event as crisis appears to favor specific analytic technologies, poised for quick judgement. Expertise in crisis management has burgeoned since the 1980s in response to large-scale industrial and environmental disasters (Jasanoff, 1994; Power, 2007) – and now pandemic disease. Such apprehension of a rapidly evolving new threat, within a short decision time, needs to be distinguished from long-term risk assessment and ‘preparedness’ for potential hazards (Lakoff, 2017). A crisis should be differentiated also from a ‘state of emergency’, which often follows a special temporality, implying a limited interlude (Fassin and Pandolfi, 2010). Emergencies usually are seen as unpredictable and brief exceptions to the normal order, a temporary suspension, with the expectation of return to normality, whereas a crisis may be prolonged and generally implies a decisive shift to a new normal. Similarly, crisis can be distinguished from ‘catastrophe’, which, as Stengers (2015 [2009]) notes, means a disruption from which there is no recovery, and therefore no demand for a decision. Accordingly, defining a phenomenon as a *crisis* will mobilize characteristic analytic technologies with distinct temporal and spatial parameters.

As Roitman (2001) suggests, declaration of a crisis not only ‘serves the practice of unveiling latencies’ in real time, it also implies judgment of what is being revealed through quantitative analysis, a deciphering of the new object of knowledge that pushes us insistently in one direction or another. ‘The point is to observe crisis as a blind spot, and hence to apprehend the ways in which it regulates narrative constructions, the ways in which it allows certain questions to be asked while others are foreclosed’ (Roitman, 2014: 94). In thus impelling us toward a norm, even a new normal, crisis management in an objective guise rapidly comes to generate new, and specifically modern, subject positions (Anderson, 2013). The crisis, as Mbembe and Roitman (1995: 323) point out, is ‘a constitutive site of particular forms of subjectivity’.

Let me emphasize here that statistical translation and encoding of the Covid-19 crisis – and quantifying the effects of various possible interventions into it – have so far failed to exhaust the significance of the event (see also Shrum et al., 2020). Rather, such refiguring of our predicament serves both to elucidate or open a window onto the situation and, using Koselleck’s words, ‘to reduce the room for maneuver’. It seems to me that expanding the room to maneuver might be a worthy task for pandemic critique. Mbembe and Roitman (1995) entreat us to resist or displace the particular forms of subjectivity dramatized and normalized in crisis, appealing to extraneous ‘lived experience’ to restrain the ‘structuring idiom’. They seek to apprehend ‘the series of operations in and through which people weave their existence in incoherence, uncertainty, instability and discontinuity’ (p. 325). They suggest we view ‘the crisis not as a system, but as a prosaic: the routinization of a *register of improvisations* lived as such by people’ (p. 326). I have reservations about submitting to the wisdom of lived experience (Scott, 1991) at a time of rampant superstition, fear mongering, prejudice, stigmatizing and hate – but I understand, too, the need to recognize social and cultural logics, and ecological settings, often erased from the conceptualization and management of crises, such as the current pandemic. We must try to imagine how Covid-19 might be figured or assembled otherwise, reconstituted as a more heterogeneous object of knowledge, as a different and more encompassing moment of truth – not simply as a measured telos delivering us a new normal.

## Making disease models

More a ratio than a biological factor, R_0_ or just R is now commonplace in calculations of epidemic magnitude and control.^6^ A function of environmental conditions and human behavior, the number R_0_ indicates the infections expected to be generated by one case in a susceptible population. Imported into epidemiology during Cold War enthusiasm for global disease surveillance and epidemic intelligence, the measure has come to articulate the severity and course of the current crisis, thereby shaping our lives in the pandemic, telling us when to lock down and socially distance or when to ease restrictions and unmask. It shows us whether social interventions are working to ‘flatten the curve’, and whether we might achieve control of the virus or even its elimination. Derived in part from studies of non-human animals, such as mosquitos, rabbits, cattle, sheep and poultry, R_0_ is deemed essential to how we comprehend the abstraction of disease among human populations. It provides the authorized answer to the question: What is an epidemic?

Mathematical modelling has only recently come to dominate the analysis of epidemics. Some public health experts, trained in conventional methods of testing, contact tracing, and isolation or quarantine of carriers, have expressed disquiet that unaccountable modelers, often schooled narrowly in statistics or physical sciences, have taken over defining the crisis. It is particularly unsettling when the models suddenly change – through new data inputs or new analytic guidelines – therefore demanding decisive action at short notice. Australia’s chief medical officer, for example, went to bed the night before the Melbourne Grand Prix in March, assured it was safe to run the motor race – only on waking early to be told by the Doherty Institute modelers that inputs or parameters had changed overnight, requiring the event to be canceled within hours. Unlike many other jurisdictions, Australia fastidiously followed the science and submitted to modelling, so stopped it was (Murphy, 2020). The dominance of modelling in framing and narrating the crisis seems to have few precedents, like so much else occurring since the beginning of 2020. The closest antecedents are probably the 20th-century profusion of economic models (Boumans, 2005; Mackenzie, 2006; Morgan, 2012); the increasingly pervasive, if not persuasive, rise of metrics in global health (Adams, 2016); and the controversial modelling of climate change, which has been criticized as the undifferentiated, alienated and ‘unassailable view from everywhere’ (Hulme, 2020: 559; see Edwards, 1999, 2011; Taylor, 1997). There can be few other examples where models with such obscure predicates and designs have become so publicly compelling and conceptually hegemonic – and such influential instruments of governance in a crisis.

‘There is probably no more legitimate use of the instrument of statistics’, wrote nineteenth-century British public health expert Arthur Ransome, ‘than its application to the study of epidemic diseases’ (Ransome, 1868: 386). After demonstrating in 1897 that mosquitoes transmit malaria parasites, tropical medicine pioneer Ronald Ross (1905, 1910, 1915) conducted a series of statistical studies of malaria control, introducing quantitative modelling into epidemiology, which he called ‘pathometry’ (Anderson, 2006; Heesterbeek and Dietz, 1996; Kucharski, 2020; Smith et al., 2012). An ecological – one might say ‘multispecies’ – mindset informed his investigation of transmission dynamics and control, though the models lacked sociological complexity, depending instead on facile racial typologies to explain human behavior. Like so many others in tropical medicine, Ross became obsessed with insect-human interactions – with measuring malaria transmission entomologically, by mosquito density – at the expense of any nuanced understanding of the sociality and cultures of human populations. In the early-twentieth century, Alfred J Lotka, a statistician for the Metropolitan Life Insurance Company, elaborated on Ross’s disease models, introducing from demography (i.e. from human population science) the notion of ‘net reproduction rate per generation’ or ‘net fertility’, a fundamentally Malthusian parameter (Lotka, 1923, 1925). In the 1950s, George Macdonald, the director of the Ross Institute of the London School of Hygiene and Tropical Medicine, also would turn his attention to the mathematical theory of malaria transmission, on the eve of the international eradication program. Macdonald borrowed Lotka’s demographic concept of the basic reproduction ratio for the disease, calling it Z_0_, meaning the number of hosts in a susceptible population expected to be infected by a single already infected host. Macdonald (1952, 1955) focused on the benefits of DDT in reducing mosquito transmission, in lowering what later became known as R_0_. Disease modelling is thus a story of how mosquitoes briefly became humans, and humans lastingly became mosquitoes. Rather than entering a more-than-human world, we were modeled into, or snared in, a less-than-human net.

It was not until the late 1970s that Robert M May and Roy M Anderson at Oxford and then Imperial College, London, along with Klaus Dietz, began to popularize the concept of the intrinsic or basic reproduction number and the accompanying symbol R_0_, applying it to other infectious diseases (Anderson and May, 1982b, 1991; May, 1981; also, Dietz, 1988, 1995). ‘The first question that ecologists ask of a virus’, May could write in 1993, ‘is what is its basic reproductive rate, what is its fitness?’ (p. 58). The priority of this question was largely the result of his statistical analyses during the previous decade. Trained as a theoretical physicist, May had reoriented his research toward ‘ecological’ modelling while working with population biologist Robert H MacArthur at Princeton. In the late 1970s, he joined Anderson, a British parasitologist, in overhauling the quantitative study of interactions of disease agents and their hosts. Around 1982, they both realized that ‘R_0_ is central to an understanding both of the epidemiology of infectious diseases and of the impact of control policies’ (Anderson and May, 1982a: 1056; Bailey, 1975). The primary determinants of a respiratory virus’s intrinsic reproductive rate appeared to be the agent’s transmissibility, the density of the susceptible population, and the frequency of contacts within it. Later, May and Anderson (1987) struggled to calculate the R of HIV, which like other sexually transmitted diseases was not density dependent, instead calling for what appeared to be unattainable sociological documentation of variation in sexual activity. At the time, human social and cultural data defied direct computation. ‘It is easy to build epidemiological models of arbitrary complexity, which may appear beguilingly realistic’, May and Anderson (1987: 140) admitted, ‘but we think there is little point in constructing them until more is known about the relevant … parameters.’

For May – later president of the Royal Society of London and Britain’s chief scientist – the ‘real hero’ of ecological modelling of viral diseases was microbiologist Frank Fenner, based at the Australian National University. With ecologist Francis Ratcliffe, Fenner in the 1950s had examined quantitatively the co-evolution of rabbit resistance and myxomatosis virulence when the virus was unleashed in vulnerable populations along the Murray River in southeastern Australia (Fenner and Ratcliffe, 1965; see Anderson, 2017). This managed outbreak constituted an unparalleled experiment in field epidemiology – and to this day represents an unrivaled modelling of epizootic disease. The ‘moral’ of this research, as May (1993: 63) later remarked, was that ‘the co-evolutionary trajectories pursued by virus-host associations, and more generally by most pathogen-host associations, involve complicated trade-offs between virulence, transmissibility, and host resistance’ (p. 64). At the same time, the research of Fenner and Ratcliffe – their ‘epidemic intelligence’ – was saturated with Cold-War concerns about risk, surveillance, infiltration, and containment, framed within a rationality of biosecurity and shaped by contemporary threats of biological warfare (Dando, 1999; Fearnley, 2010). Fenner’s dead rabbits thus could be represented as casualties of the Cold War. The myxomatosis epizootic among Australian rabbits became the model for understanding later human epidemics, including even the emergence of AIDS. But in recognizing such ecological homologies, epidemiologists effectively were reimagining human societies and communities as elementary biological collectives or herds (Erickson and Mitman, 2007). What might be lost and what gained in a crisis when human populations are remodeled as rabbit vermin? What critical decisions and judgments are rendered permissible, even necessary?

In other words, this has become a story of how quantitative modelling of disease outbreaks – arising from imperial and settler colonial situations – might embed connections with other animal collectives, more-than-human relations, while conventionally discounting or ignoring human sociality and cultural difference, thereby refiguring *Demos* simply as *Zoe*. In a sense, then, when Latour (2020) demands more ecological insight and Kirksey (2020) recommends multispecies perspectives in our apprehensions of the Covid-19 pandemic, they may be missing the point. Disease modelling already gathers and connects species, but generally as asocial and unstructured, even anomic, collectivities – one might even say as bare life (Agamben, 1998, 2020a, 2020b; Anderson, 2020a). According to epidemiologist Gideon Meyerowitz-Katz (2020), ‘Even the best, most sophisticated models only take the first steps in the tangled web of interconnectivity that we call society.’ It is not a matter of substituting sociology for ecology, but rather the need to recognize the heterogeneity of collectives and the diversity of relations within and between them, thereby deriving more intricate, realistic, and inclusive ecological reasoning – to which most disease modelers have so far proven resistant.

## Crisis in the herd

British authorities took up disease modelling with alacrity during the epizootic of bovine spongiform encephalopathy (BSE) – the ‘mad-cow’ crisis – of the 1990s. Since 1985, farmers in southern England had been reporting strange behavior in their cattle, with many beasts losing weight, staggering, falling down and dying. Postmortem examination showed surprisingly spongy brains, resembling those from sheep that died from scrapie and from humans who succumbed to kuru or Creutzfeldt-Jakob Disease (CJD). All of these diseases are infectious, and uniformly fatal, though at the time the causes were still enigmatic: some medical scientists attributed them to a ‘slow virus’, alluding to the presumed agent’s long incubation period, while others suspected a misshapen transmissible protein, called a prion. The BSE epizootic had been propagated through industrial feeding of contaminated meat and bone meal to herbivore cattle. By 1990, tens of thousands of the national herd were afflicted; at the end of the decade, almost two hundred thousand had perished. Within a few years, it was reported that scores of Britons were developing a new variant of CJD, the likely consequence of abnormal prion exposure through beef consumption (Anderson, 2019). Roy Anderson and colleagues from Oxford’s Department of Zoology – including a young theoretical physicist, Neil M Ferguson – set about modelling the spread of the disease among cattle, hoping to judge the most effective method of culling the herd and thus curtailing the epizootic (Anderson et al., 1996).^7^ They sought to calculate R_0_, the prion’s basic reproduction number, which, they wrote with the fervor of converts, ‘characterizes the rate of growth of an epidemic, gives insight into the proportion of the population which will be affected once the disease becomes endemic, and determines the criteria for disease elimination’ (Ferguson et al., 1999: 31; also Donnelly and Ferguson, 2000). They shrugged off vexing issues such as spatial clustering in a disease outbreak, admitting that ‘the models used assume homogeneous mixing and exposure of the Great Britain cattle population’ (p. 31).

Just as the BSE epizootic was waning, Britain confronted another outbreak, this time foot-and-mouth disease among pigs, sheep, and cattle. First detected in southern England, the virus spread rapidly across the nation, boosted by close contact between animals as they moved across country. Lodged then at Imperial College, London, Anderson and Ferguson again led efforts to model the new outbreak. Their predictive epidemiological modelling again was predicated on assumptions of a homogeneous animal population distributed across uniform space, a schema lacking any stochastic or random elements and omitting any specific transmission route parameters (Ferguson et al., 2001). But all models, according to critics, are necessarily facile ‘abstractions of complex processes’ (Bickerstaff and Simmons, 2004: 400), and the ones from Imperial College at least had the advantage over more complicated versions of quickness, when dispatch was of the essence. ‘The statistical imperative of bringing the R_0_ value below one … became the dominant rhetorical tool in scientific and political debate around the management of risk’ (Bickerstaff and Simmons, 2004: 402). Achievement of an R_0_ less than one, thereby deflecting the crisis, seemed to demand an immediate cull of all susceptible animals near any infected farm, which resulted in the slaughter of almost six million sheep and cattle by the end of the year. Many veterinarians, arguing from experience and tacit knowledge of farming practices, found themselves struggling to relate these deterministic models to what they were observing on the ground. To counter statistical abstraction, they proposed an alternative situated scientific analysis, dependent on understanding differences in species immunity, local spatial practices and complex patterns of interaction and relationality between animals. But abstract predictive modelling proved too powerful a political machine. As Law (2008) reflects, ‘a simple but opaque epidemiological technology was used to draw an unnecessarily alarmist line through the animal collective’.^8^

Emboldened by success in modelling epizootics among farm animals, Ferguson turned his attention to possible viral pandemics. A particularly potent strain of influenza A virus, H5N1, emerged in China around 2003, spreading rapidly through wild birds and poultry, and occasionally crossing over to humans with lethal impact. By 2005, the virus was widespread among avian populations across Southeast Asia. Although no sustained human-to-human transmission had occurred, virologists feared that mutation someday might make H5N1 more readily transmissible, perhaps creating a pandemic. Ferguson and colleagues in London therefore developed a simulation model of influenza transmission in Thailand in order to evaluate the effectiveness of mass use of prophylactic antiviral drugs and regulations that would diminish community contact rates. Using techniques that accounted for outbreaks among British herds, the modelers determined that targeted prophylaxis along with what they were now calling ‘social distancing’ should together reduce R_0_ to a level that might staunch the disease’s spread among humans (Ferguson et al., 2005).^9^ Confident in generalizing from Thailand to the region, their study blithely ignored local social structures, customs, habits and livestock management practices – focusing instead on the contribution of human population density, or the frequency of social contact, to contagiousness. During a crisis, Ferguson and his colleagues protested, ‘decisions have to be made before definitive information was available on the severity, transmissibility, or natural history of the new virus’ (Lipsitch et al., 2009: 112).^10^ In contrast, Asian researchers were describing in ethnographic detail the ecological and sociological specificities affecting potential viral transmission in the region. Interested in the geo-spatial distribution of H5N1 in Thailand, they recognized the importance of free grazing ducks, associated with local rice crops during particular seasons, in explaining the spread and persistence of the influenza virus (Fearnley, 2020; Gilbert et al., 2006; Keck, 2020). But as Kleinman and other medical anthropologists lament, most discussions of H5N1, to the contrary, ‘all too often consist of allegations of blame and assumptions of cultural shortcomings rather than of serious investigations of the political, cultural and socioeconomic realities of the societies that have come to be associated with the virus’ (Kleinman et al., 2008: 1). They propose a ‘biosocial’ approach, which would allow ‘deeper understanding of the inseparable interactions that take place among biological, social, political and environmental phenomena’ (p. 3). But how to make time for such nuanced inquiry in a crisis? This is, of course, the regulatory question that framing an event as a crisis will generate.

## Emergence of the novel coronavirus

According to Camus (1947), a few seemingly trivial incidents, scarcely noticed at the time, signaled the onset of plague in the town of Oran. But in early 2020, British epidemiologists and public health officers knew well in advance exactly what was about to strike them. For months they had been reading reports of a novel coronavirus emerging and ramifying from Wuhan, China, stealthily spreading around the world. By early February, SARS-CoV-2 was infiltrating communities throughout Europe. At the beginning of March, disease modelers told Britain’s Scientific Advisory Group for Emergencies (SAGE) that sustained transmission of the new virus was inevitable.^11^ Some public health experts recommended the government step up testing, contact tracing and isolation of those positive – but soon it was clear that the necessary reagents and kits and infrastructure were lacking. Neil Ferguson recalled that he had stopped modelling strategies based on widespread testing around January 28, when he learned there was not enough equipment to carry them out (Taylor, 2020). Instead, drawing inferences from past influenza epidemics, SAGE initially decided to attempt to mitigate, rather than suppress, the coronavirus. It determined to take measures, including social distancing and hand hygiene, that might lessen R_0_ a little, flattening the curve on graphs of the virus’s incidence. ‘Our aim is to try to reduce the peak, broaden the peak, not suppress it completely,’ said Patrick Vallance, the government’s chief scientific advisor, on March 3 (Conn et al., 2020). The goal was ‘to build up some kind of herd immunity’ – meaning that enough people would eventually be exposed to the virus, and rendered immune, to limit or stop transmission.^12^

But these plans went awry very quickly after a revised model from Imperial College was delivered to SAGE on March 16, at the start of the perceived crisis (Ferguson et al., 2020).^13^ Observing the high case mortality rates and imposition on intensive care facilities in northern Italy, Ferguson and his colleagues had recalculated the likely effects of mitigation efforts in Britain, finding that hundreds of thousands of deaths would still occur and the National Health Service would collapse – long before any herd immunity was attained. Using the coding devised to assess strategies against avian influenza in Thailand, the modelers figured that even a combination of mitigation options – including case isolation, voluntary home quarantine, social distancing, and intermittent school closures – might lead to a national catastrophe. They therefore concluded that ‘mitigation is unlikely to be a viable option without overwhelming healthcare systems, suppression is likely necessary in countries able to implement the intensive controls required’ (Ferguson et al., 2020: 10). Until a vaccine could be distributed, a lockdown was imperative in order to reduce R_0_ below one, in accordance with the revised model indicating that ‘epidemic suppression is the only viable strategy at the current time’ (p. 16). Yet Ferguson and his colleagues warned that ‘no public health intervention with such disruptive effects on society has previously been attempted for such a long duration of time’ (p. 16). Soon after reading the report, Prime Minister Boris Johnson began awkwardly to change tack, lurching toward mechanisms to suppress the virus. The model proved a decisive, even critical, arbiter. When Britain ‘locked down’ on March 23, there had been, cumulatively, 798 confirmed cases of Covid-19 and eight deaths. By the end of April, more than 160,000 Britons had tested positive and at least 21,000 had died from the coronavirus, but the curve slowly was flattening. Perhaps rashly, Johnson then decided to ease restrictions (Conn et al., 2020).

The limited parameters and the simplifications of the Imperial College modelling made many experts uneasy. Stephen Eubank, director of the Biocomplexity Institute at the University of Virginia, and colleagues expressed disappointment with expedient recourse to the ‘workhorse’ compartmental model, which partitions populations into susceptible, infected, and recovered or removed categories (the SIR model), specifying transmission rates between them. ‘The model’s reliance on a simplified picture of social interactions limits its extensibility,’ they concluded. When alternative network and individual-based models, so much more adept at capturing heterogeneity, are available, ‘why are we still using models developed 15-20 years ago?’ (Eubank et al., 2020).^14^ The concept of contact or social interaction in Ferguson’s model appeared misleadingly facile, lacking in context, as if there is no such thing as society. It resembled a regnant Thatcherite knowledge regime in epidemiology, a decisive rejection of social heterogeneity and complexity (Hall, 1979). To compensate for scanty sociology, there were calls to incorporate real-time contact data from social media and the internet, geo-tagged for location, using predictive algorithms (Carey, 2020a, 2020b). Others instead demanded ‘full and frank disclosure’ of embedded assumptions, uncertainties, and normative values. They warned against ‘excess complexity,’ ‘spurious precision,’ and the ‘questionable appearance of rigour’ (Saltelli et al., 2020: 484). According to Andrea Saltelli and colleagues, ‘once a number takes centre-stage with a crisp narrative, other possible explanations and estimates can disappear from view’ (p. 483). They claimed that ‘good modelling cannot be done by modellers alone. It is a social activity’ (p. 484).

The principle of deploying abstract models to shape decision making in a crisis elicited more general criticism. Some condemned reliance on modelling and displacement of ‘old-fashioned’ epidemic control techniques such as surveillance and testing, contact tracing, and isolation of confirmed cases – even though the epidemic in Britain had long ago outpaced the capacity to roll out such procedures. The editor of the *Lancet*, Richard Horton (2020), deprecated the stacking of SAGE with statisticians and behavioral scientists – the latter fixated on information processing and communication strategies – and the exclusion of conventional public health expertise. ‘There is evidence on modelling and on behavioural science,’ Horton said in April, ‘but I don’t see the evidence from the public health community or from the clinical community’ (Boseley, 2020). According to British pediatrician Anthony Costello, ‘the basic public health approach is playing second fiddle to mathematical modelling’ (Boseley, 2020). The authority of these abstractions, the presumed power of such reductionist techniques – supposedly paradigmatic of ‘good science’ – evoked considerable concern, even scorn. ‘To decipher the patterns of the virus, its consequences and how best to address its spread,’ writes Littoz-Monnet (2020), ‘policymakers reverted to knowledge produced through models and simulations, deflecting their horizons away from past or present realities towards the realm of “virtual” scenarios played out by modelling studies.’ Problems of governance shriveled into those sanitary solutions modeled as necessary in a crisis. ‘This automatically resulted in the exclusion of alternative forms of expertise, and the avoidance of broader debates with the public.’ Or as a medical anthropologist from the Harvard Medical School asserted, ‘bourgeois empiricists build fable models whose assumptions are usually conjured from the standpoint of dominant interests’ (Richardson, 2020).

## The model crisis

Regardless of any sweeping criticism, modelling has proven a compelling mode of anticipatory governance (Aykut et al., 2019; Guston, 2014; Michaels, 2017) during the Covid-19 crisis, allying a sense of urgency in judgment with creditable legerdemain of scientific technique. While I have focused here on British modelling, because its failures and contestations have been so vividly expressed publicly, I could have adduced similar examples in multiple other national jurisdictions, including continental Europe, North America, and Australasia. According to Rhodes and colleagues (2020: 253), ‘COVID-19 is coming to be known in maths and models…. Mathematical models and projections have become ubiquitous.’ Emerging at ‘a moment of actuarial saturation,’ statistical models or simulations help to simplify decision making in exigent and complex conditions, substituting in effect the ‘speculative forecast’ for the messy sciences of the actual (Adams et al., 2009: 247). Modelling makes certain population policies and subject positions conceivable, even necessary, at the same time as it aims to ‘depoliticize’ debate, attempting to silence other voices and alternative imaginations of the future, further reducing room to maneuver in a crisis and enacting a performance of calculation and control. Once an obscure epidemiological enthusiasm, models these days are ‘lived as *anticipated potentials*, affecting actions, publics and policies *in-the-now*’ (Rhodes et al., 2020: 253) – thereby epitomizing modernity. In thus being treated as a herd, people find that the scope of social life, its range of potentialities, gets constricted and homogenized; and they feel ever more alienated and excluded from decision making. To be sure, we may need to model our way out of a crisis, to make abstraction work for us, to get busy with the pandemic – but must our models be so circumscribed and limited, their performance so inhibiting? As Wynne (2010: 301) writes of climate models, critique ‘is about understanding their *conditional* validity, and the implicit diverse meanings and alternative potential trajectories … deeply embedded in them and their framing’. Or as Taylor (1992: 141; also, Taylor, 1989) proposes, since models are not just purporting to represent social worlds but now work to intervene in them, we should solicit a critical expansion of ‘the boundaries of the ecology of agents and resources implicated’.

Another means to voice such concerns involves reflecting on the function of *configuration* in explanation of disease outbreaks. Rosenberg (1993; Anderson, 2010) distinguishes between epidemiological theories premised on either structurally complex ‘configuration’ or linear ‘contamination’. During the past century, there were a few public health experts who have sought to understand intricate and heterogeneous configurations or assemblages of epidemic disease, whether drawing from sociology and political economy or from ecology and biology. These elite epidemiologists try to identify underlying structures and dynamic modifications in our societies and environments that promote or retard the emergence and spread of infectious diseases. Meanwhile, most others on the front line have tended to default to serviceable, unadorned assumptions about contamination, diligently culturing microbes and tracing their passage through populations. These ‘microbe hunters’ are absorbed in detecting contacts, tracking disease incidence, indifferent for the most part to situation or context.

Rosenberg (1993) observes that the successes of germ theories early in the twentieth century, and the ‘heroism’ of utilitarian microbe hunters, never entirely eliminated configurational inclinations among microbiologists and epidemiologists. Social medicine, the integrative and multifactorial explanatory framework derived from sociology, with its distinctive emphasis on the etiological influence of structural inequalities in human societies, developed in the nineteenth century and continues to exert influence through the political economy of health (Farmer, 2001; Rosen, 1947). Disease ecology, gleaned from biological and environmental sciences, was increasingly invoked after the 1918–19 influenza pandemic to account for infectious disease emergence and amplification, positing disequilibrium of interactions between host and parasite, or the micro-evolution of animal resistance and microbial virulence, as well as direct effects of the physical environment, as instigators of the rise and fall of outbreaks (Anderson, 2004; Morse, 1993). This was the theoretical apparatus of Ross and Fenner, but not until the 1980s, with the emergence of new epidemics such as AIDS, did concepts of disease ecology truly proliferate, coalescing into the principal framework for grasping how microbes locate different niches to colonize. Although there have been some exercises in ecological analysis of the advent of Covid-19, often perfunctory and frequently stigmatizing particular communities, the modelling of the current pandemic has mostly resorted to conceits of contamination, suppositions of contact without context, cartoons rather than simulacra, instead of recognizing and reconstructing more complex and heterogenous structures in which the virus thrives. The places where we congregate thus wither into plain and meager spaces of contamination, shorn of any sociological and ecological diversity (Anderson, 2020b; see Sismondo, 2020). This is what it means to perform modelling in the current crisis.

Such disparate visions of contamination and configuration evoke different politics of life. What sorts of population or herd may be brought into being through operation of a crisis technology like modelling? The statistical model or simulation surely exemplifies what Foucault (2009: 10) called ‘the apparatus (*dispositif*) of security’, developed during the past two centuries in response to ‘epidemics and the medical campaigns that try to halt epidemic or endemic phenomena’. The securitizing apparatus is constituted through calculation and analysis and regulation, transforming the normal into a statistical distribution around a norm, conjuring a milieu in which other mechanisms of biopower, whether juridico-legal or disciplinary, might exert effects on a population. In Foucault’s schema, security is a way of making ‘the old armatures of law and discipline’ (p. 10) function in organizing a *collective*, not just in punishing or transforming a singular body or subject. Evidently, security responses to Covid-19 have involved centrifugal expansion of juridical processes, which punish and exclude, as well as disciplinary strategies, which codify what is forbidden and permitted in daily life, drilling and correcting populations (Foucault, 1991 [1975]). Models or *dispositifs* predicated on simple contamination, bruiting the danger of contact without context, would seem to generate more coercive or repressive force fields of biopower, commanding, for example, lockdown, curfew and border closure, measures involving the suspension of rights. Whereas modelling more sensitive to ecological and sociological configuration – to complex and particularistic assemblage – would appear to necessitate and to enable disciplinary measures, reaching more deeply into the social body, changing conduct and the sense of the self, reforming customs and habits, and adapting a population to the new normal.^15^ The different premises and parameters for modelling the crisis, for reducing R_0_, can thus produce distinct subjectivities and collectivities – sometimes a diverging, and then again sometimes a composite, politics of life.

To engage the problematics of apprehending a pandemic surely requires critical inquiry, in the sense that ‘critique’ and ‘critical’ derive from the same root as crisis. Passing judgment on the merits of something is what we must do in a crisis. Foucault (1997: 47) associated critique with ‘the art of voluntary insubordination, that of reflected intractability’, and therefore represented it as a form of epistemic virtue. But it seems timely here to return critique to its pre-Enlightenment ethos, to recuperate it as the art of making decisions or distinctions, of deliberation – not simply the art of not being governed. As Brown (2005: 5) argues, we need to reunite critique and crisis, recognizing a critical condition as ‘an urgent call for knowledge, deliberation, judgment, and action to stave off catastrophe.’ She urges ‘the depiction of critique as non-optional in the restoration of an organism’s or polity’s health.’ Similarly, Latour (2004: 237) attempts to rework critique through ‘the merging of matters of fact into highly complex, historically situated, richly diverse matters of concern’. For him, ‘the critic is not one who debunks, but one who assembles’ (p. 246). I am old enough to recall the same protestations and remonstrances in the 1980s during the AIDS pandemic, another crisis, to be sure, but one rarely compared to our current predicament for reasons unclear to me. The difference then was that we were asking how to have *theory* in an epidemic. According to Treichler (1999: 9), the critical response to AIDS was ‘the struggle for an intelligent vision to live by in the face of crisis, contradiction, and the urgent need to make life-or-death decisions’.

One could translate that struggle into another question in another era: How can we have critical theory in the Covid-19 pandemic? That is, how might Covid-19 be refigured or assembled otherwise? Can critique reconstitute the crisis as a more encompassing and ambient subject of knowledge, affording room to maneuver? Can it socialize or de-herd or ecologize or even queer our models? This is, of course, a reiteration of Treichler’s quest to undertake critical theory in relation to the AIDS epidemic. She was making a plea for ‘the careful examination of language and culture that enables us, as members of intersecting social constellations, to think carefully about ideas in the midst of a crisis’ (Treichler, 1999: 1). She was seeking a broader ‘epidemiology of signification’, an eclectic, accessible, and responsive epidemiological remodelling, which might incorporate complexity, difference, otherness (Treichler, 1987). It was critical to ‘understand how various kinds of knowledge are produced, the rules and universes of discourse through which truth is variously represented and understood’ (Treichler, 1999: 10). In *Impure Science*, Epstein (1996) shows how activists reimagined AIDS in the 1980s, often persuading scientists and epidemiologists to share, or at least acknowledge, their sociological, and sometimes ecological, visions. They were ‘yoking together moral (or political) arguments and methodological (or epistemological) arguments’ (p. 336) and conducting a ‘sustained lay invasion of the domain of scientific fact-making’ (p. 330). This was not a matter of debunking or unmasking the sciences – rather it was a form of critical care, a means of tending to their impoverished and wasted condition, feeding them up. ^16^ It was critique as Latour (2004) advises, a gathering or assembling of ever more subjects and participants, the crafting of matters of concern.

Treichler’s question of theory was a deliberate echo of Douglas Crimp’s (1987b) provocation: ‘How to have promiscuity in an epidemic’. He was alluding to the multiplying of relations, connections, liaisons, and impacts – a pro-creation as much intellectual or cognitive as sexual. For Crimp in the 1980s, AIDS was not only a time for mourning and reflection, it also presented an opportunity – an ethical juncture – for self-expression and self-assertion and militancy (Crimp, 1987b, 1989). The pandemic, he wrote, ‘intersects with and requires a critical rethinking of all of culture: of language and representation, of science and medicine, of health and illness, of sex and death, of the public and private realms’ (Crimp, 1987a: 15).^17^ So, too, does Covid-19, more or less. Silence equals death, as we used to say. Recall ACT UP – the AIDS Coalition to Unleash Power, which was inspiring Crimp’s reflections on how to have promiscuity in an epidemic. Or, just get busy with the abstraction of a pandemic, as Camus once proffered. Perhaps, then, we could start with some critical promiscuity.

## Notes

1. ‘The more banal, the more commonplace, the more predictable, the triter, the staler, the dumber, the better’, as DeLillo (1991: 9) puts it. Or rather, perhaps, banal in Morris’s (1988: 26) sense of ‘an irritant that returns again and again to trouble cultural theory’.

2. In the original: ‘Mais quand l’abstraction se met à vous tuer, il faut bien s’occuper de l’abstraction’ (Camus, 1947: 92).

3. Žižek’s (2010: 95) looser translation is: ‘The old world is dying and the new world struggles to be born; now is the time of monsters’.

4. Auguste Comte (1969 [1830–1842]: 325) argued that sociology existed to help us comprehend crises. See also Jacob Burckhardt (1964).

5. Wiener and Kahn (1962), Erikson (1968) and Kuhn (1962) are a few prominent examples.

6. I mostly refer here to R_0_, the basic reproduction number, as the measure of how transferable a disease is, but strictly this should be used only when the whole population is susceptible to infection. Where there are individuals no longer susceptible, whether through immunity or other protection, then R, the effective reproduction number, is more apt. In order to control the spread of disease R_0_ or R should be reduced to less than one.

7. As a physics graduate student, Ferguson had been inspired to shift to epidemiology when he heard May lecturing. The influence of physicists, like May and Ferguson, on disease modeling may have diminished the ecological and sociological complexity of the simulations.

8. The development of later pandemic models from these studies of the national herd may partly explain the peculiar territoriality of responses to Covid-19, especially the national, rather than global, delimiting of the problem.

9. The term ‘social distancing’, derived from social psychology, seems to have crossed over into epidemiology around the turn of the last century, when it spread wildly. The influenza modeling was supported by the National Institutes of Health’s Models of Infectious Disease Agent Study (MIDAS), established in 2004 after anthrax and smallpox terrorism scares in the United States, with the remit to develop mathematical and computational models of potential disease outbreaks: see Halloran et al. (2008).

10. For simulation of an influenza pandemic in Britain and the United States, which recommended case isolation and household quarantine, while advising against border closure and travel restriction, see Ferguson et al. (2006).

11. Chris Whitty, the chief medical officer, and Patrick Vallance, the government’s chief scientific advisor, chaired SAGE, which included Ferguson until his resignation in May 2020 after flouting distancing rules. See Stewart (2020).

12. The concept of ‘herd immunity’ was translated from veterinary practice to human public health in the 1920s, in reference to the resistance to infection of a human collective; see Topley and Wilson (1923) and Dudley (1929). The medical usage of the herd thus predates its occurrence in outbreak modeling; see Fine (1993) and Jones and Helmreich (2020). While herd immunity is achievable when the virus has low infection fatality rates, like most influenza variants, it proved unfeasible with the more lethal novel coronavirus. For the first few months of Covid-19, however, everyone persisted in fighting the war they had prepared for against a new influenza virus.

13. There were a number of alternative – though apparently less influential – models circulating at the time, including those devised by Nicholas G Davies and colleagues at the Centre for Mathematical Modelling of Infectious Disease at the London School of Hygiene and Tropical Medicine. See Davies et al. (2020).

14. In fact, it may be useful to go back even further, to look again at some of the later studies of AIDS transmission, which necessarily employed social science to describe complex patterns of contact. See Black and Singer (1987) and Garnett et al. (1996).

15. For an elaboration of this argument in the Philippine and Australian contexts, see Anderson (2020c), Maddock and Anderson (2020). See also Jasanoff (2020).

16. I mean ‘care’ in Puig de la Bellacasa’s (2011) sense of ‘giving marginalized issues a voice in the staging of technoscientific mediations’ (p. 96). At the same time, I appreciate Murphy’s (2015: 726) warning of the ‘fraught genealogies of care’, and the need to trouble and unsettle possibly hegemonic structures.

17. Critical promiscuity clearly is related to what Murphy (2017) as called ‘distributed reproduction’, an alternative apprehension or othering of biopolitics – and to Stengers’s (2005) broader proposal for an ‘ecology of practices’.
